# Monthly Summary

**Published:** 1888-11

**Authors:** 


### Monthly Summary.

EFFECTS OF MENTAL OVERWORK.—*A Common Form of Illness which is Generally Disregarded.*—Some interesting though not novel observations on the symptoms of mental fatigue were discussed at a recent meeting of the Anthropological Society. The result of these investigations goes to prove that weariness of mind, the result of work, like other forms of exhaustion, is recognizable under the two different though related aspects of irritability and incapacity. Further careful inquiry into the same subject would probably show that here, as elsewhere, the former of these conditions is introductory to the latter, and is the natural sequel to that stage of apparently successful overaction which is seen when an organ still fully capable is unduly stimulated.

The observations referred to were culled from a series of reports by school-teachers, and included details of their own sensations as well as of the children under their care. The signs of mental irritability were apparent in sleeplessness and nervous laughter; of fatigue, in sleepiness and incapacity for task work. Lolling, yawning, and a languid manner told that the will was flagging. Headache suggested overstrain in study combined with defective ventilation, and perhaps a too sparing diet; while some curious facts bearing on the causation of color-blindness and somnambulism were also noted. Thus, in one case the blue-color perception was for a time obliterated, and the sufferer from this defect found herself painting ivy leaves a bright orange; while in another a student, having retired to rest on the eve of an examination, awoke at his desk to find that he had been busily engaged in drawing humorous cartoons relating to a former conversation. Here we have an instance of cerebral irritation due to overwork which suggests a somewhat close connection between dreaming and somnambulism, and affords a clue to the physiology of the latter condition.

Overwork, both mental and bodily, is at once the most general and the least regarded form of illness to which we are liable in the present age. Do what we may, it is next to impossible to escape from it; but there is, at all events, a certain satisfaction in being able to recognize its features. We must not forget, however, that it is also to a considerable extent a preventable evil, and it is certainly a matter for satisfaction that this fact is not ignored by the reforming party in the Legislature. Its treatment in individual cases requires chiefly that due attention be paid to the two great essentials of timely rest and wholesome diet. Work, however irksome, may, it is generally allowed, be undertaken on a liberal scale if only it be not too continuous, but is broken by timely and adequate intervals of rest. The value of a plain and liberal dietary is hardly less, and we may take it as a maxim for the times that, so long as appetite and sleep are unimpaired there is no dangerous degree of overwork, and, conversely, that a failure in either of these respects should be regarded as a warning signal, to which attention should be paid by relieving the strain of exertion.—*Lancet.*

---

DO ALL AMALGAMS SHRINK?—One of our popular writers says they do. This is as great a fallacy as his assertion "that there is little difference in amalgams; they are all made very similiarly." There is as much difference in amalgams as in gold foils, and this is saying much.

We are aware that many amalgams shrink; but we are also aware that an amalgam may be so made as to expand. Why then may not the proportions of metals be so adjusted that the compound will neither shrink nor expand? That this has been the aim of many manufacturers is without question, and that some have attained this end there is no doubt.

The fact is "the trade" is in such a wretched condition that the demand for a low price is constantly tempting the manufacturer to debase his compound without regard to the result. The same author from whom we have just quoted says : " Thirty-five or forty per cent. of silver with sixty to sixty-five per cent of tin makes just as good an amalgam as any of the more expensive ; and, therefore, it matters but little what amalgam a dentist uses." Accordingly, men are found reckless enough to make such a compound, and by giving it a high sounding name, and agreeing with their dealers to give them a half of the gross returns, the profession is flooded with it, and the only boast is that " It is offered at a remarkably low price."

Then comes the anathemas against all amalgams : "I filled teeth for Judge Story's wife only two years ago, and now they are a wreck. Don't tell me amalgam won't shrink. I have now demonstrative proof that it will shrink, and that the edges will break away, and that the teeth are ruined by it."—*Items of Interest.*

---

THE GLANDS.—The functions of glands are among the most interesting and most important of the organic processes. They rank next to the vital organs in importance, and any interference with their functions is serious.

They are numerous, and may be divided into the true glands, the lymphatic or conglobate glands, the ductless glands. Names are intended to be expressive of the quality of a thing, or function of an organ. If this rule had been followed in the naming of the different glands, none but the true glands would have been entitled to the appellation of gland. But they were all called glands before their respective functions were understood, and having once found place in our literature under this name, it was not advisable to change the names of any of them, but to designate them according to their respective functions. Certainly those organs which elaborate and give forth all the amazing varieties of animal juices, which, notwithstanding their variety, are all drawn from the same source, should be called glands. These are the true glands, and though the microscope shows scarcely a difference in the form and structure of the cells composing their tissues, yet the diversity of their secretions is something marvelous. There is no saliva in the blood, no gastric juice, no milk, no semen. The elements of these substances, even to the life spirit of the seminal germ, are in the blood, but the secretions themselves are elaborated in the glands by a cell action peculiar to each variety.—*Western Dental Journal.*

---

CIGARETTE SMOKING.—Prof. Dudley (*Med. News*) rejects the popularly held opinion that the baneful effect of cigarette smoking is due to the adulteration of the tobacco with noxious drugs, and by experiments on mice shows conclusively that the toxic agent is carbonic monoxide, which, however, results alike from the combustion of tobacco, whether consumed in cigarette, pipe or cigar.

A spectroscopic examination of the blood of three mice, dying after a very brief exposure to an atmosphere of cigarette smoke, showed an entire conversion of oxyhemoglobin into carb-oxy-hemoglobin, the cause of death being CO poisoning. As is well known, CO is exceedingly poisonous and in contact with blood converts the life-supporting oxyhemoglobin into the lethal carb-oxy-hemoglobin, a non oxygen-carrying compound, difficult of oxidation, which may cause death by suffocation, although there may be free entry of pure air into the lungs.

Cigarette smoking is only more harmful than cigar or pipe smoking because those addicted to the first habitually inhale the smoke, drawing into the greatest depth of the lungs the poisonous CO, the result of the combustion of the tobacco.

[A consideration of Dr. Dudley's experiments suggests that a wide difference exists between the effects of tobacco smoked and chewed, and that if the latter habit is filthier it is far less harmful. It is to be regretted that the evil resulting from smoking is not limited to the consumer of tobacco, but must extend to those who are unfortunate enough to be in the smoker's proximity. Patients should be cautioned against remaining in unventilated apartments in which smoking is going on, for the air in such places must soon become vitiated by the noxious CO. The ill effects of an atmosphere of tobacco smoke on young children and delicate females is thus explained.]—*The Polyclinic*.

---

WHERE DO TEETH DECAY?—When a dentist examines for cavities in a mouth which he has never seen, he looks in three places only: It is where the enamel dips down and forms a deep crevice,—either sulci in the top or the side of a tooth,—or makes a dimple somewhere there is no business to be a defect, he looks between the teeth, just below or above the point cf contact; and he looks at the little creases near the margin of the gum. He need not look anywhere else. Why? Because the act of mastication and talking will generally keep the food from lodging on other portions of the teeth. And where no lodgement of food takes place, and the health of the patient does not cause excessive acidity of the oral fluids, there is no decay of the teeth. I know a man, fifty-three years of age, whose teeth are perfect in form and arrangement, though they never have been brushed; only one cavity, and that was where a dentist had used a file. He has simply washed them with water after every meal, yet he has a clean mouth. Now,some of you may remember a skull which I showed you, of a child six years of age, where the twelfth-year molars were not erupted, and yet all four were carious; cavities that no human power other than a filling for each could have prevented from involving their speedy and entire destruction had the child lived.. No possible brushing could have kept them clean.—*Items of Interest.*

---

A WONDERFUL GERMICIDE.—A simple germicide prepared by Dr. J. D. Fernandez. is very efficacious. It is a composition of sodic hypo-sulphites, diluted sulphuric acid and spirits of lavender, and it is certainly most beneficial.

Mr. Dillon, before he was first taken ill, directed the doctor to prepare something for the telegraph operators. The doctor prepared this, and the telegraph operators have been using it regularly now for four weeks or over. Half a teaspoonful, night and morning, is the dose. Since then not a man in the operating room who has used this germicide has been taken sick. The telephone operators in the room over the telegraph office, by some misunderstanding, did not get any of the germicide, and all but one have been taken down. Two of the clerks in Mr. Dillon's office failed to take it, and they are both ill. Mrs. Dillon arrived here from Pablo on the day that Mr. Dillon was taken ill, and she nursed him through his sickness, being by him all the while. She took the germicide regularly, and hasn't been troubled with yellow fever at all. D. J. Crowley, manager of the office, nursed his wife during her sickness until her death, using the medicine punctually, and he escaped the fever.—*Jacksonville Despatch to N. Y. World.*

---

CAUSES OF MORTALITY.—Dr. Drysdale brings forward many facts to show that indigence is a potent cause of the high death rate in the civilized nations of Euorpe. He proves in many ways that insufficient food, clothing and shelter combine to raise the death rate far above what it is in those classes or countries living under more favorable conditions. Instead, therefore, of governments offering prizes and premiums upon large families, he would propose that they should put a fine upon any one, rich or poor, who in an old country produced more than four children.—*The Polyclinic.*

---

DETECTION OF CONCEALED INSANITY BY THE USE OF NITROUS OXIDE.—Hamilton relates cases in which patients, who carefully concealed their delusions, were made to display them fully while under the influence of nitrous oxide. He believes the gas to be very useful, also as a therapeutic measure in various mental disturbances, by effecting a diversion of morbid thought, or a temporary suspension of memory.—*S. Cal. Practitioner.*

